# Compressive torsional hysteresis performance of concrete filled square CFRP steel tube

**DOI:** 10.1038/s41598-025-98032-w

**Published:** 2025-05-14

**Authors:** Peng Kuan, Wang Qing-li

**Affiliations:** 1 School of Intelligent Manufacturing, Chengdu Technological University, Chengdu, 610031 People’s Republic of China; 2 School of Civil Engineering, University of Science and Technology Liaoning, Anshan, 114051 People’s Republic of China

**Keywords:** CFRP-steel tube, In-filled concrete, Compressive-torsional hysteresis loading, Numerical study, Bearing capacity, Civil engineering, Mechanical engineering

## Abstract

To study the mechanical performance of concrete-filled square CFRP steel tube under compressive-torsional hysteresis loads, 9 concrete-filled square CFRP steel tube to analyze the failure mode, torque angle curve, triaxial strain, and the synergistic effect of steel tube and CFRP on the specimen under cycle loading. The experimental results show that the steel tube and CFRP can work together. Based on the tests, a numerical simulation method is firstly proposed to estimate the concrete-filled CFRP steel tube compressive-torsional specimens under hysteresis loading, and then validated against the representative tests results. As the axial compression ratio increases(0 < *n* ≤ 0.45), the torsional displacement of the specimen is constrained, resulting in increase of bearing capacity By contrast, as n continuous increases, the initial stiffness of the component begins to decrease, and the bearing capacity also decreases when *n* > 0.45. In addition, the increase in steel content, CFRP layer, and specimen material strength has a significant impact on the bearing capacity and initial stiffness.

## Introduction

Concrete filled steel tube (CFST) is a new composite structure composed of steel tube and concrete, two most commonly used building materials. Under the action of load, the steel pipe and concrete can give full play to their respective advantages and maintain good coordination, that is, good filling effect of concrete can delay the local buckling of steel and improve the overall stability of the structure^1–6^. Meanwhile, the steel pipe has a good constraint on the concrete, making the concrete in a triaxial compression state and improving the strength and plasticity of concrete. Therefore, concrete filled steel tubes are widely used in large structures such as bridges, high-rise buildings, transmission towers and wind turbines. However, concrete filled steel tubes still have some shortcomings in some aspects, that is, concrete filled steel tubes are easy to be corroded in seawater or corrosive soil, which affects the bearing capacity and durability of the structure, and is easy to occur local buckling in the process of bearing load^7–9^. CFRP is widely used in building engineering due to its advantages of light weight, high strength and good corrosion resistance^10–12^. CFRP is closely adhered to the outer surface of concrete filled steel tube, which can be combined into a new composite structure—CFRP—concrete filled steel tube. This new structure has the advantages of high bearing capacity, good durability, reduced use of steel and reduced component weight^13–19^.

In recent years, CFRP—concrete-filled steel tubes (CFST) as a research hotspot have been extensively studied at home and abroad. At present, the research contents mainly include the mechanical properties, corrosion resistance and fire resistance of CFRP—concrete-filled steel tubes. Tang et al. studied the axial compressive bearing capacity of concrete filled with stainless steel tubes confined by CFRP^20^, with 24 short columns in total. The test parameters include the number of CFRP layers and the thickness of stainless steel tubes. It is found that the typical axial load–displacement curve has four stages: elastic stage, secondary rising stage, repeated fracture stage and post-fracture stage; The increase of bearing capacity is roughly linear with the increase of CFRP layers. Zhang et al. carried out the axial compression test of FRP—concrete-filled steel tube column under cyclic load. There are 6 short columns in total. The test parameters include the type and thickness of FRP^16^. It is found that when the axial strain develops to 0.02, the ratio approaches 1.0, and the plastic strain is difficult to recover. Carbon fibres play a more important role in inhibiting the development of plastic deformation than basalt fibres (BFRP). Park et al. conducted compression-behysteresis tests on existing square concrete filled steel tubes and square CFRP—concrete filled steel tubes^13^. The test parameters include concrete strength grade and CFRP layer number. The test results show that the increase of the number of CFRP layers only increases the bearing capacity slightly, but the increase of the number of CFRP layers greatly improves the ductility of the test piece, and the increase of the number of CFRP layers slows the local buckling of the steel tube at the bottom of the test piece. Tao et al. studied the fire resistance of FRP—concrete-filled steel tube^21^, obtained the failure mode after fire through the fire resistance test of circular FRP—concrete-filled steel tube axial compression test piece, and analyzed the section temperature, axial deformation and the fire resistance of the test piece. The research results show that if FRP—concrete-filled steel tube can be reasonably designed, the required fire resistance limit can be reached. Dong et al. carried out the corrosion test of CFRP—concrete-filled steel pipe pile in high humidity environment^22^, 12 test pieces in total, the size of test pieces is 114 mm in diameter and 1200 mm in height. Under different corrosion levels, the half-cell potential, corrosion products, corrosion expansion and adhesion were studied. The test results show that the mechanical property and corrosion resistance of concrete filled steel pipe are significantly improved when the concrete filled steel pipe is bonded with CFRP sheet. The CFRP—concrete filled steel pipe pile is an effective method to protect the pile from corrosion.

At present, there are few studies on the coupled load of CFRP—concrete-filled steel tubes, especially the bottom cycle reciprocating load. Therefore, this study determines the main influence factors on the test piece, i.e. axial compression ratio and the addition of different amounts of carbon fiber reinforced polymer layers. The mechanical properties under the action of low cycle reciprocating compression torsional hysteresis loop load are studied. Combined with the function of ABAQUS software, the mechanical property change of each material in the process of loading is analyzed to replicate the *T*-*θ* behavior observed in the physical sample. Using insights gained from practical experiments and computational simulations.

## Specimen preparation and experimental design

### Specimen preparation

**Fig. 1:**
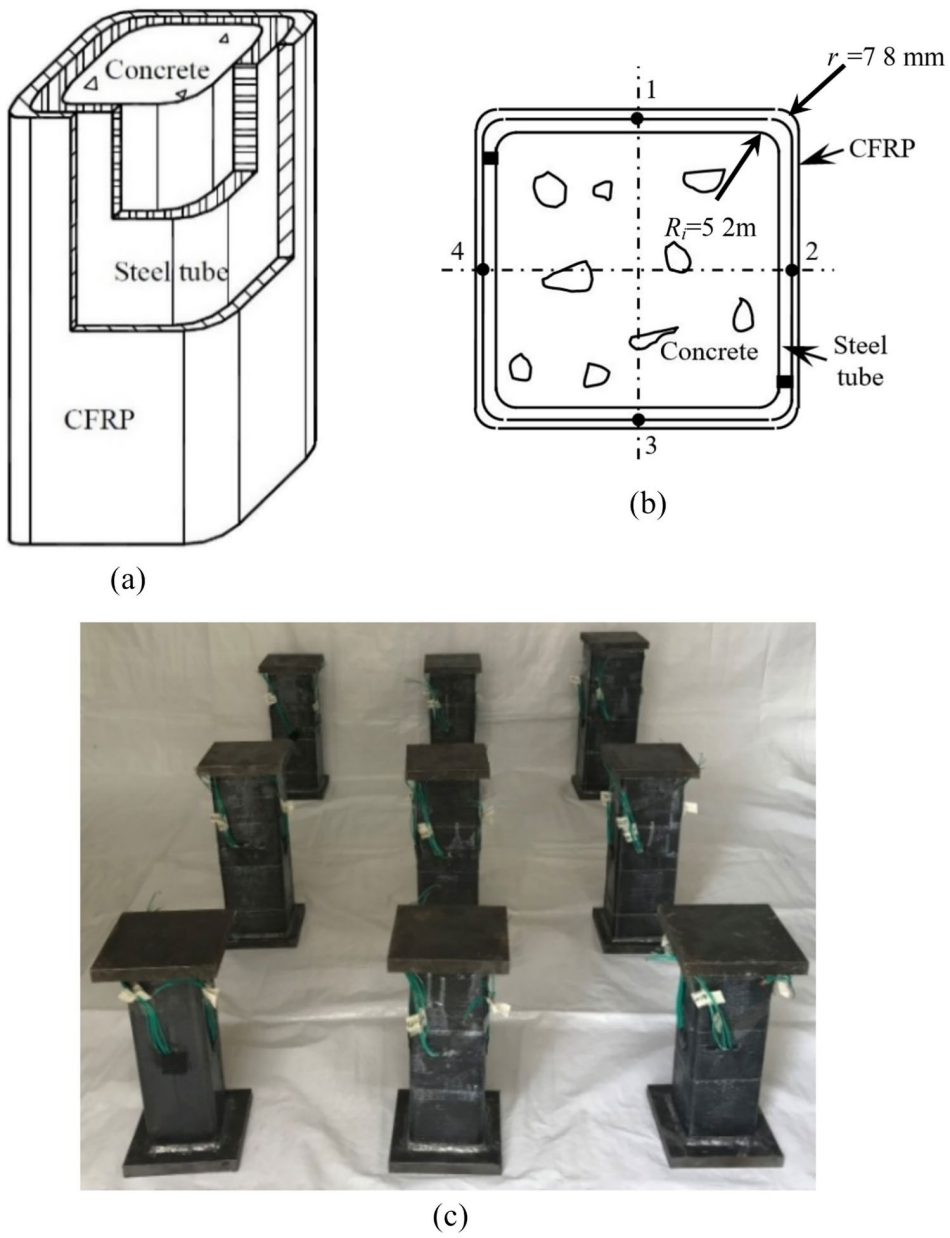
Schematic diagram and physical image of the cross-section of the specimen. (**a**) Sample composition diagram of specimen, (**b**) Cross section of specimen, (**c**) All compressive-shear specimens after preparation.

A total of 9 concrete-filled square CFRP steel tube specimens are designed for the compressive-torsional hysteretic test. The *L* of all specimens is 360 mm, *B*_s_ is 120 mm, *t*_s_ is 2.6 mm, the inner corner (*R*_i_) and outer radii (*r*_o_) are 5.2 mm and 7.8 mm, respectively. The axial compression ratio *n* and the number of layers of transverse CFRP are the main research factors for concrete-filled square CFRP steel tube compressive-torsional specimens. Other parameters are shown in Table 1. All specimens after preparation is shown in Fig. 1.

**Table 1:** Other parameters of compressive-shear specimens.

Serial number	No	*n*	*m*_t_	*N*_u_/kN	*N*_0_/kN	*ξ*_cf_
1	SCTH30A	0.3	0	100	2.76	1
2	SCTH31A	0.3	1	100	2.86	2
3	SCTH32A	0.3	2	100	2.96	3
4	SCTH01B	0	1	120	4.45	4
5	SCTH11B	0.15	1	120	3.89	5
6	SCTH31B	0.3	1	120	3.09	6
7	SCTH41B	0.45	1	120	2.59	7
8	SCTH61B	0.6	1	120	1.58	8
9	SCTH31C	0.3	1	140	3.21	9

### Material properties

The characteristics of the steel pipes used are detailed in Table 2.

**Table 2:** Performance of steel tubes used in C-CF-CFRP-ST specimens.

*f*_y_/MPa	*f*_u_/MPa	*E*_s_/GPa	*v*_s_	*ε*^′^/%
466	610	206	0.28	27

The concrete’s composition is meticulously outlined in Table 3.

**Table 3:** Mix proportion of concrete used for C-CF-CFRP-ST specimens.

C	FA	S	G	W	SP
0.6	0.4	2	1.4	0.35	0.01

The carbon fiber used is woven by Japanese Toray. The main performance indexes of carbon fiber are shown in Table 4.

**Table 4:** Main mechanical property indexes of CFRP.

Thickness (mm)	*E*_cf_ (GPa)	*ε*_cftr_ (me)	*ε*_cflr_ (me)
0.111	230	3000	3000

## Test phenomena and damages

**Fig. 2:**
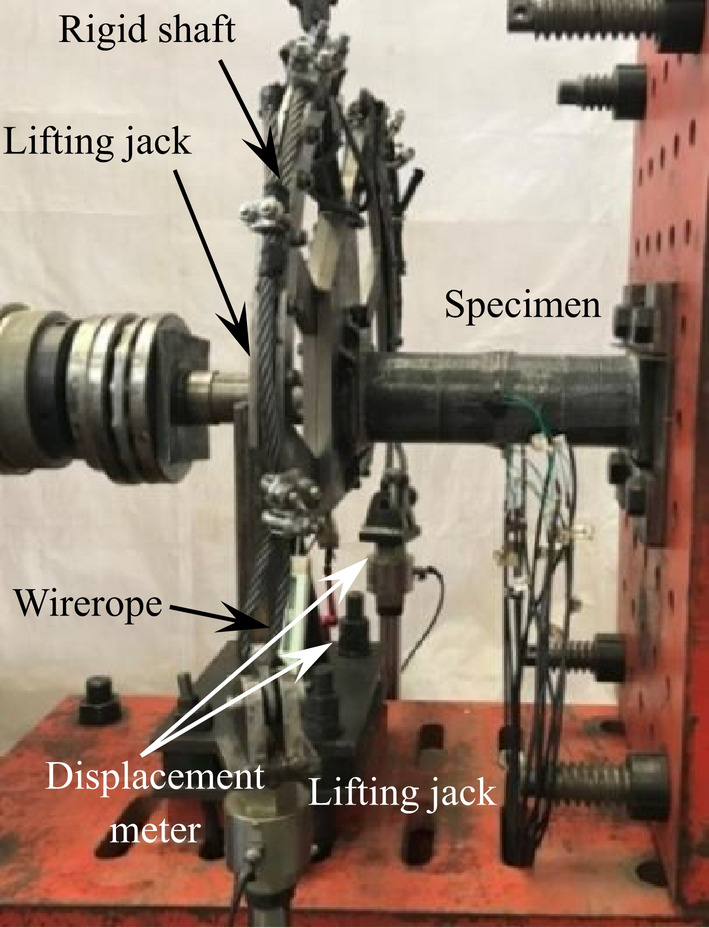
Setup for the compressive-torsional hysteresis test.

**Fig. 3:**
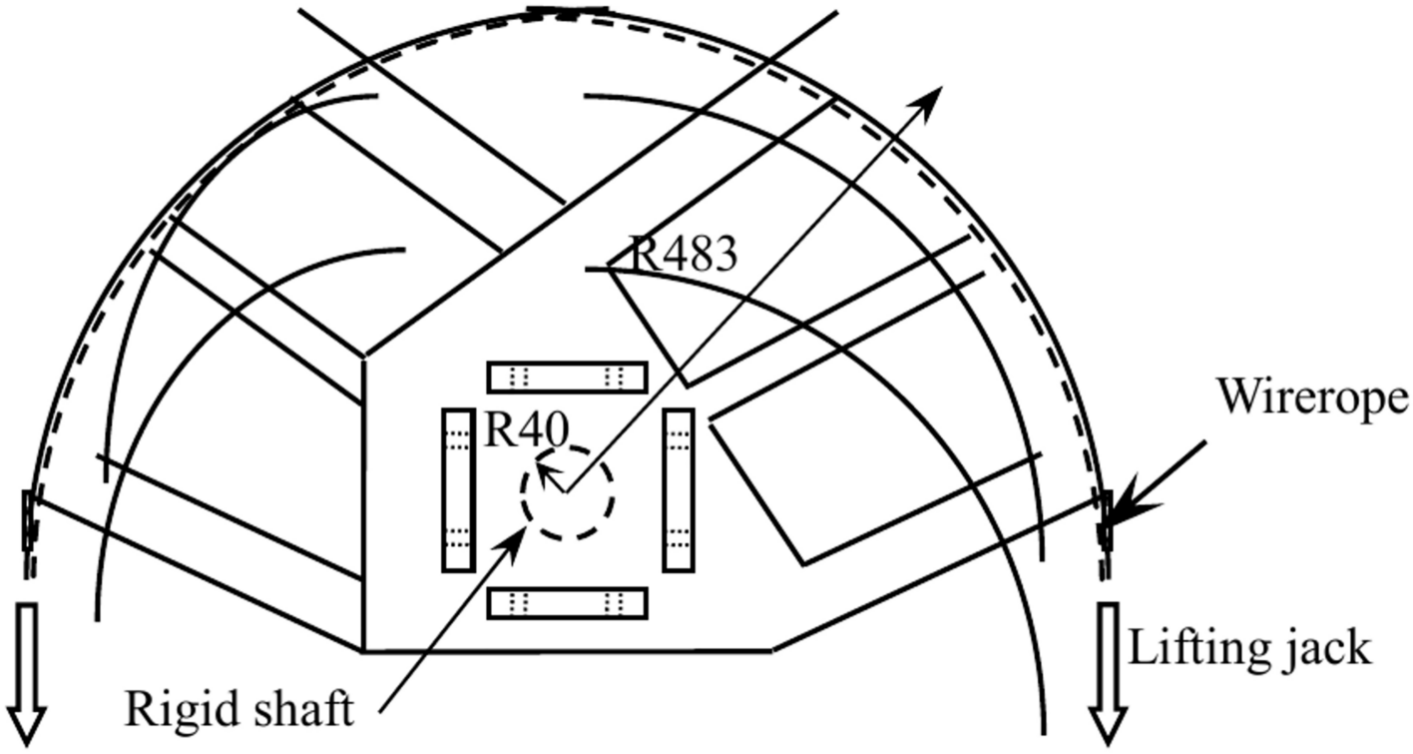
Sample composition diagram of steel arm.

Figure 2 shows the setup for the compressive-torsional hysteresis test. On the basis of the compressive-torsional static performance test setup of previous design^23^, the other side of the steel arm (Fig. 3) is added and connected to a 200kN hydraulic jack through a wirerope. The end plate on one side of the specimen is fixed to the embedding device as a fixed end, and the end plate on the other side is fixed to the steel arm as a loading end. During the experimental loading, a 2000kN hydraulic jack is used to apply axial loading, and the jack on one side of the steel arm pulls the wirerope to load the torque. After loading to the specified torque or angle, the wirerope is unloaded, and then the other side jack is used to pull the wirerope. The above steps are repeated to achieve the purpose of hysteresis loading.

The test adopts loading-displacement control method for loading. Loading control and graded loading are adopted in the initial stage, with loading of 0.25 *T*_uc_ (Estimated bearing torque), 0.5 *T*_uc_, and 0.7*T*_uc_ respectively, and each level of loading is cycled twice; Afterwards, displacement control and graded loading are adopted, with loading of 1.0 Δ_θ_ (The torsional angle corresponding to the yield of steel), 1.5 Δ_θ_, 2.0 Δ_θ_, 3.0 Δ_θ_, 5.0 Δ_θ_, and 7.0 Δ_θ_. The first three levels of displacement are cycled 3 times per level, and the remaining levels of displacement are cycled 2 times per level. Before the experiment, perform a preload on both sides, with a loading value of 30% of *T*_y_. Standard of stop the test is: (1) The displacement ductility coefficient reaches 8 (i.e. the angle control is loaded to 8 Δ_θ_); (2) The loading drops to 40–60% of the peak load; (3) The displacement is close to the range of the actuator.

## Experimental curves

**Fig. 4:**
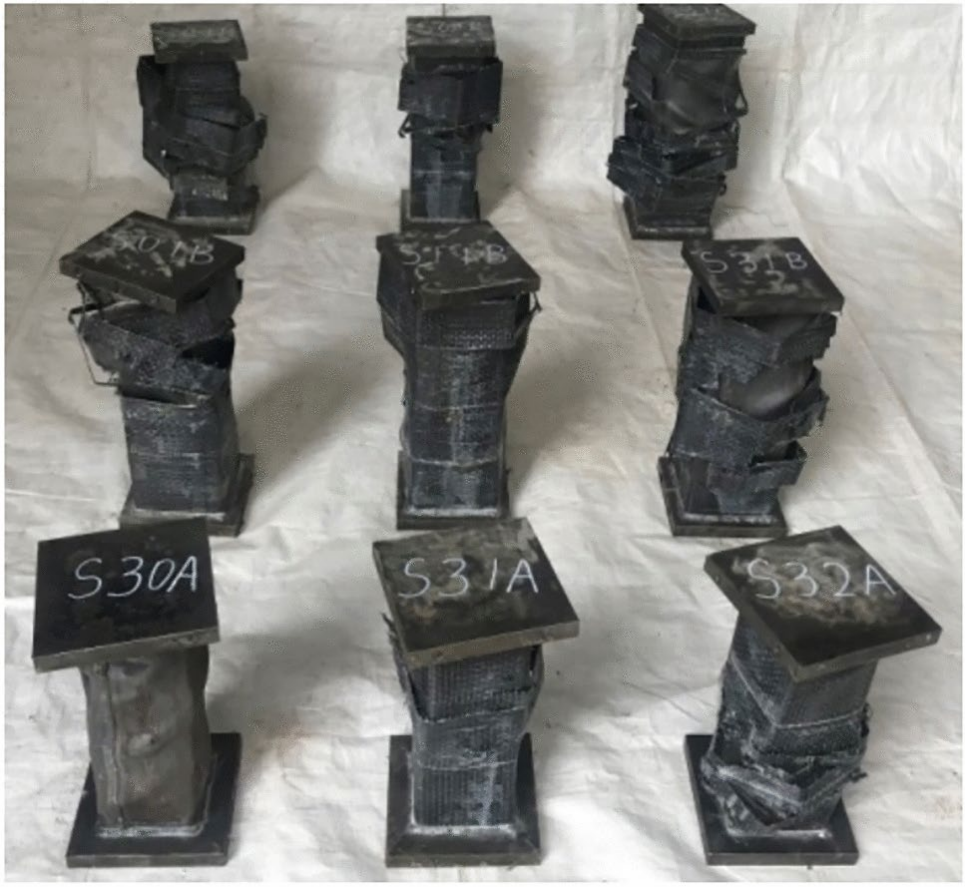
All concrete-filled square CFRP steel tube compressive-torsional specimens under hysteresis loading after test.

Figure 4 shows all concrete-filled square CFRP steel tube compressive-torsional specimens under hysteresis loading after the experiment. In order to better demonstrate the failure modes and characteristics of the specimens, some representative specimens are selected for display.

### *T*-*θ* curve

1. *T*-*θ* Hysteresis curve

**Fig. 5:**
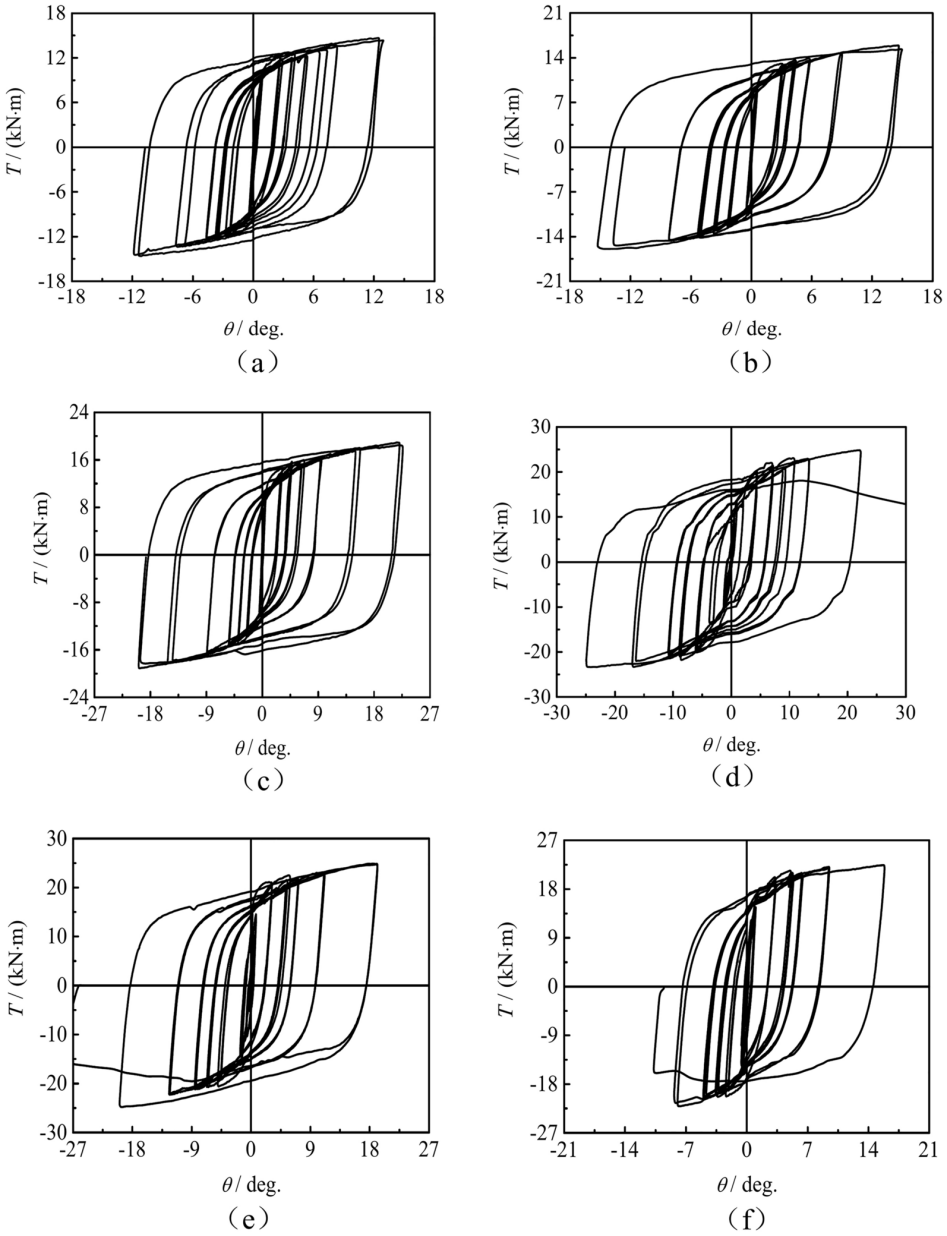

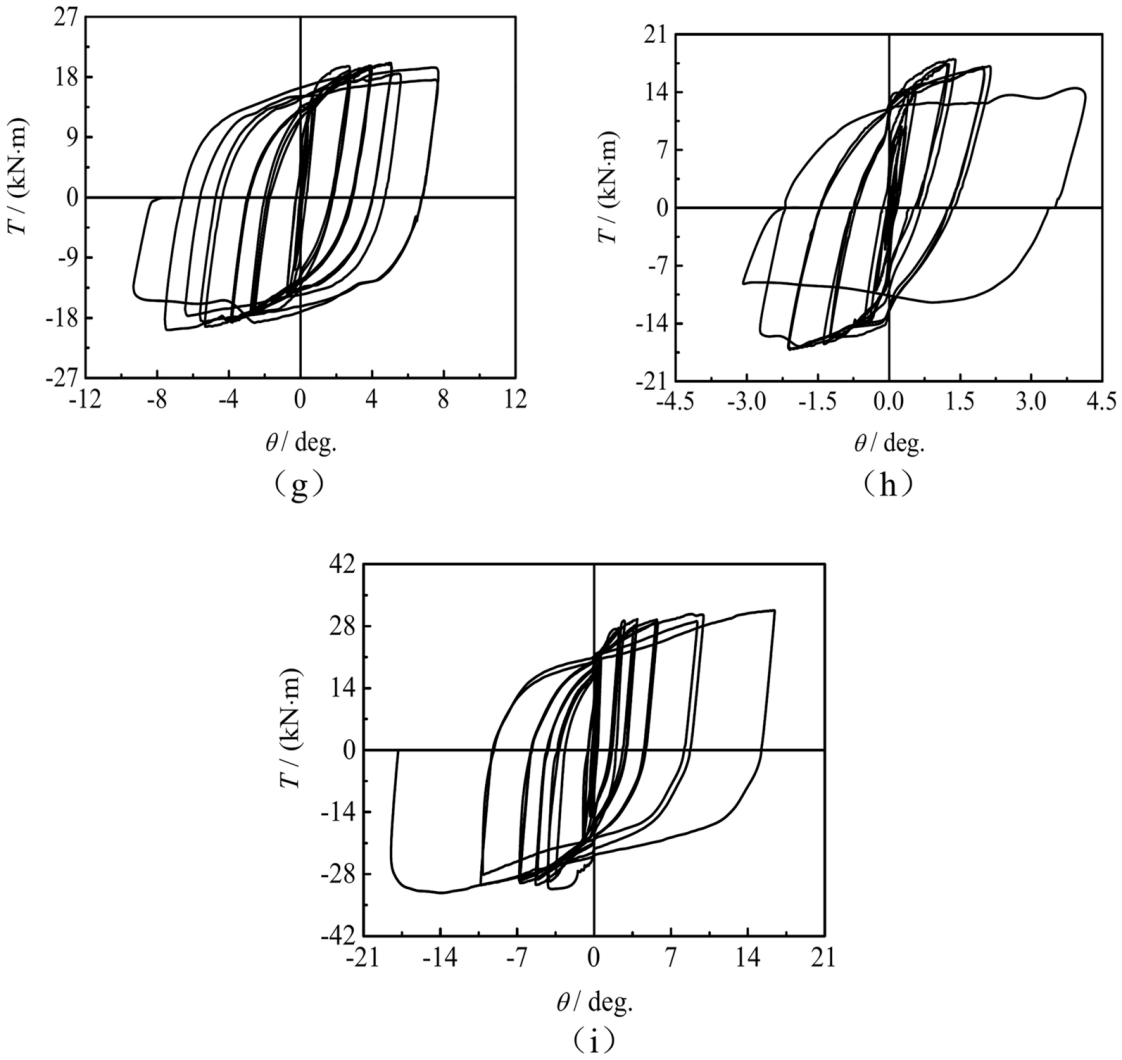
*T*-*θ* hysteresis curves of the square section specimens. (**a**) SCTH30A, (**b**) SCTH31A, (**c**) SCTH32A, (**d**) SCTH01B, (**e**) SCTH11B, (**f**) SCTH31B, (**g**) SCTH41B, (**h**) SCTH61B, (**i**) SCTH31C.

Figures 5 shows the *T*-*θ* hysteresis curves of concrete-filled square CFRP steel tube compressive-torsional specimens under hysteresis loading. It can be seen that the hysteresis curves are spindle shaped and relatively full, without any pinching phenomenon. The hysteresis curve approximately shows a linear variation in initial stage of loading. Stiffness of the specimens gradually decreases after yielding, and the stiffness of the specimen remains basically unchanged during the process from unloading to reverse loading.

(2) *T*-*θ* Skeleton curve

**Fig. 6:**
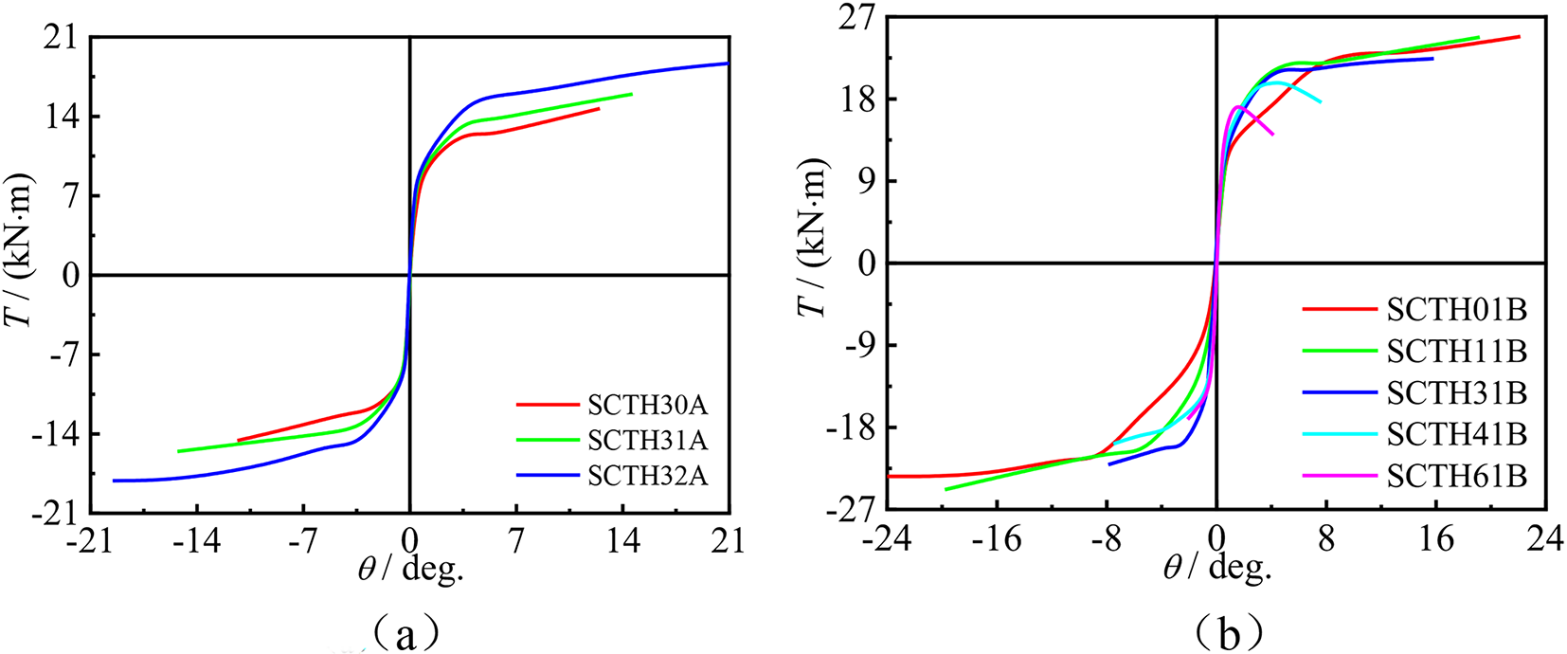
The *T*-*θ* skeleton curves of the square section specimens. (**a**) *n* = 0.3, *D*_s_ = 100 mm, (**b**) *m*_t_ = 1, D_s_ = 120 mm.

In order to more clearly reveal the influence of test parameters on concrete-filled square CFRP steel tube compressive-torsional specimens under hysteresis loading, skeleton curves is proposed based on the hysteresis curve. Figure 6 shows the *T*-*θ* skeleton curves of the all specimens. It can be seen that the curves of specimens with *n* < 0.45 No descending trend. The bearing capacity of the specimens increases with the increase of *m*_t_, but there is no significant change in the stiffness during the elastic stage. There is no significant change in the stiffness of the specimen during the elastic stage as *n* increases, and the bearing capacity of the specimen first increases and then decreases.

### Three directional strain

**Fig. 7:**
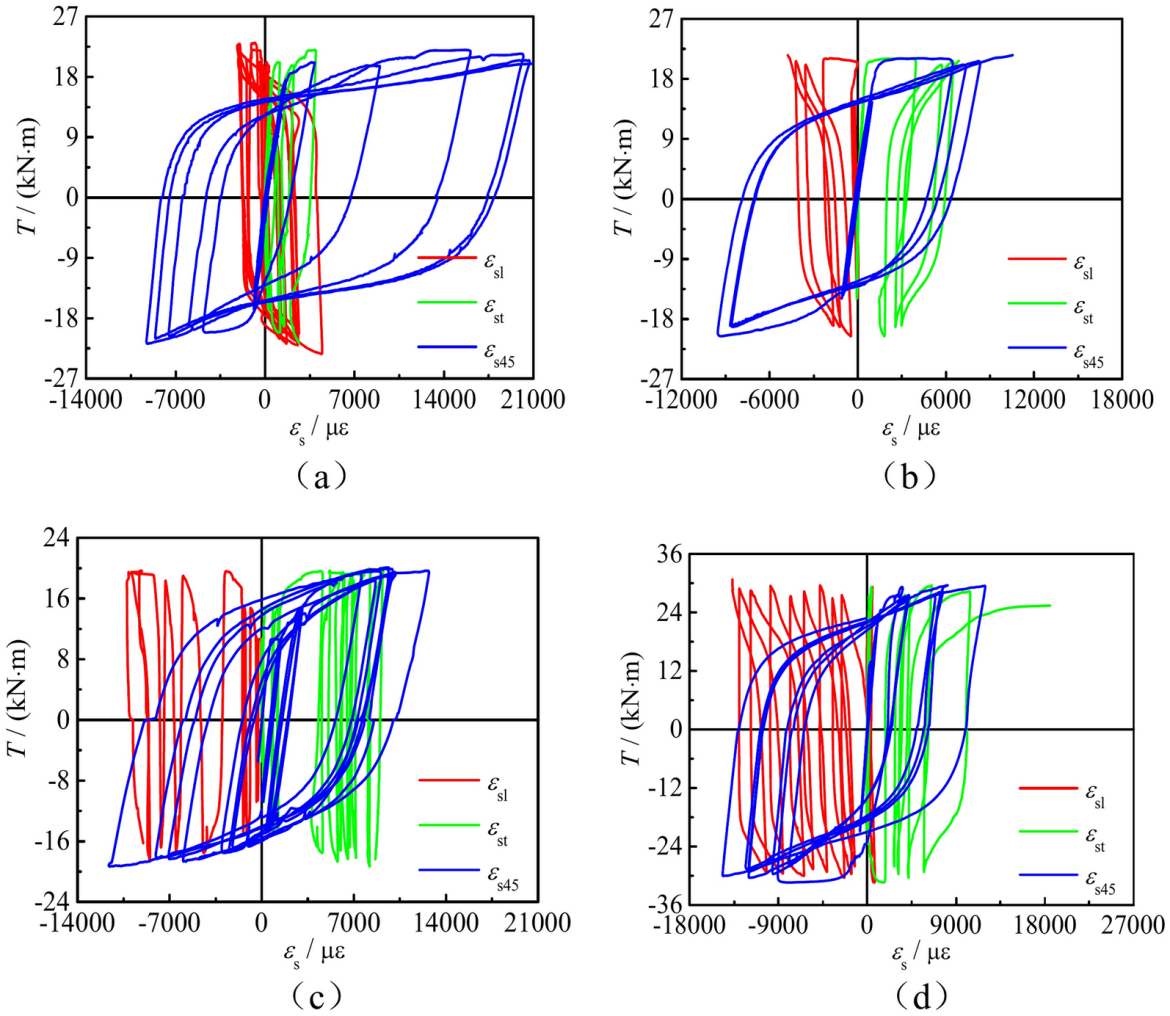
*T*-*ε*_s_ curves of partial specimens under hysteresis loading. (**a**) SCTH01B, (**b**) SCTH11B, (**c**) SCTH41B, (**d**) SCTH31C.

Figure 7 shows the *T*-*ε*_s_ curves of concrete-filled square CFRP steel tube compressive-torsional specimens under hysteresis loading. The positive and negative values of *ε*_45_ alternate. For specimens with *n* = 0, *ε*_sl_ and *ε*_st_ at the same point exhibit different signs and alternate positive and negative. For specimens with *n* ≠ 0, *ε*_st_ is always positive and *ε*_sl_ is always negative. This phenomenon is due to the fact that both torque and axial loading can cause deformation of the specimens. Torque plays a major role in deformation in the 45° direction, while axial loading plays a major role in transverse and longitudinal deformation.

### Collaborative work between steel tube and CFRP

**Fig. 8:**
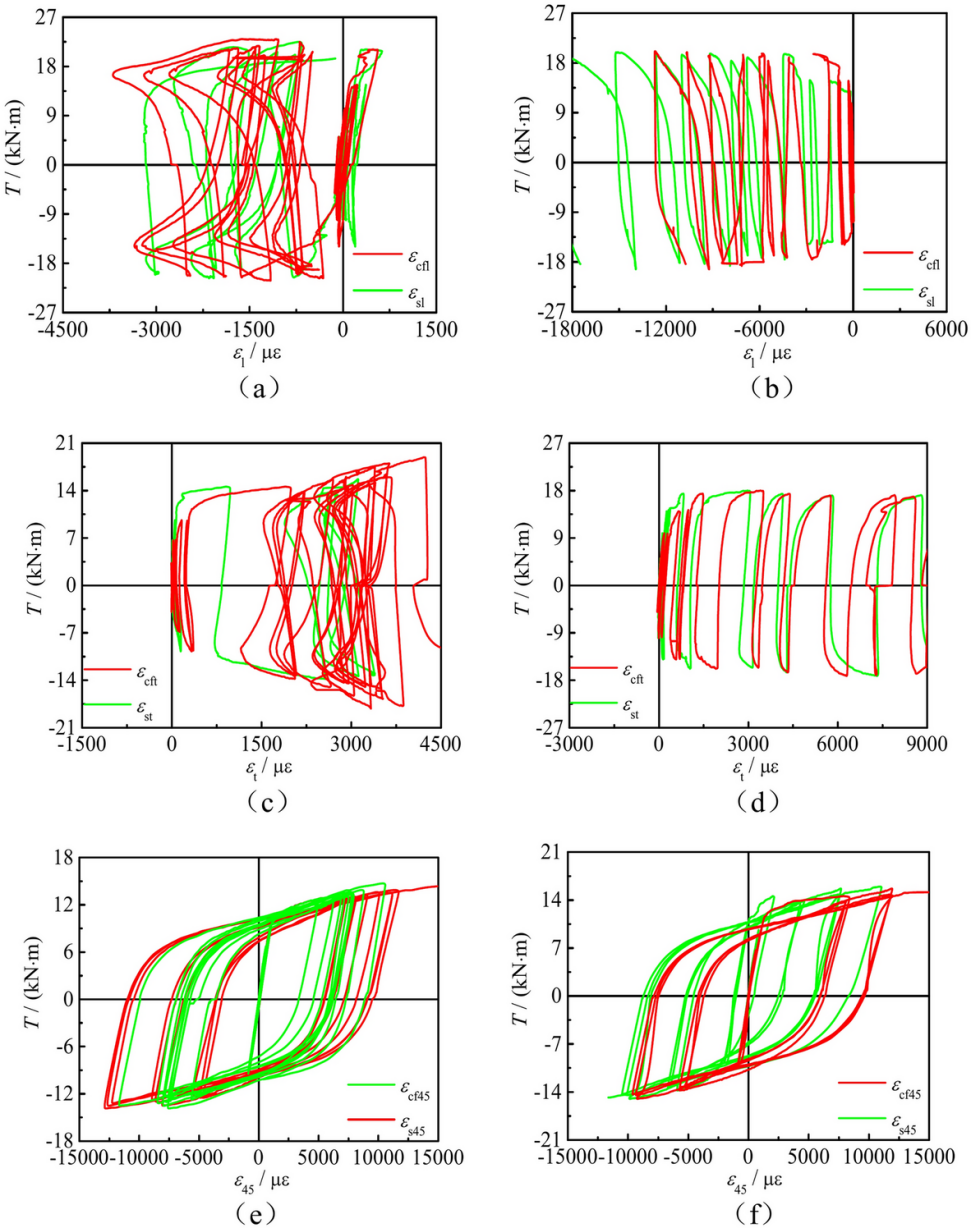
*T*-*ε* curves of steel tube and CFRP at the same position. (**a**) *T*-*ε* curve of SCTH11B, (**b**) *T*-*ε* curve of SCTH41B, (**c**) *T*-*ε* curve of SCTH32A, (**d**) *T*-*ε* curve of SCTH61B, (**e**) *T*-*ε* curve of SCTH31A, (**f**) *T*-*ε* curve of SCTH32A.

Strain gauges on steel tube and CFRP at the same position are pasted, and extract the measured stress–strain curves, as shown in Fig. 8. Those includes stress–strain curves of CFRP and steel tube in the longitudinal (*T*-*ε*_l_), transverse (*T*-*ε*_t_), and 45° (*T*-*ε*_45_) directions on concrete-filled square CFRP steel tube compressive-torsional specimens under hysteresis loading. It can be seen that the strain of the steel tube and CFRP is basically consistent in all three directions, indicating that the steel pipe and CFRP can work together under compressive-torsional specimens hysteresis loading.

### Indicator analysis

1. Stiffness

**Fig. 9:**
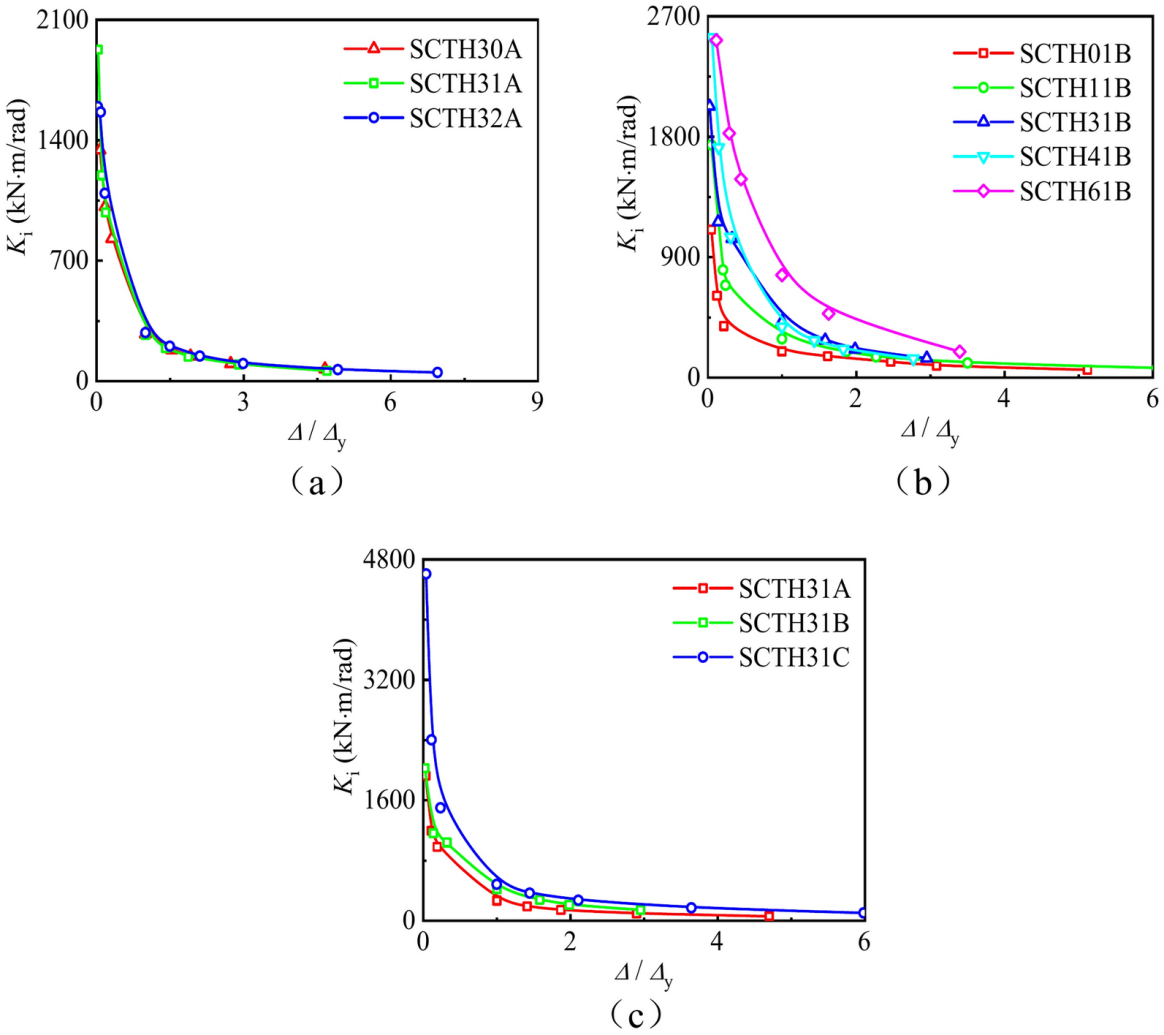
Secant stiffness curves of all specimens under hysteresis loading. (**a**) Specimens with different *m*_t_, (**b**) Specimens with different *n*.

Figure 9 shows the secant stiffness curves of concrete-filled square CFRP steel tube compressive-torsional specimens under hysteresis loading. It can be seen that the number of transverse CFRP layers has no significant effect on the stiffness of the specimen. The *n* has a significant impact on the stiffness of the specimen. The stiffness of the specimen increases with the increase of the *n* in the initial stage of loading. The transverse CFRP gradually fractures with continuous loading. Afterwards, the stiffness of the specimens gradually approaches.

(2) Energy dissipation

**Fig. 10:**
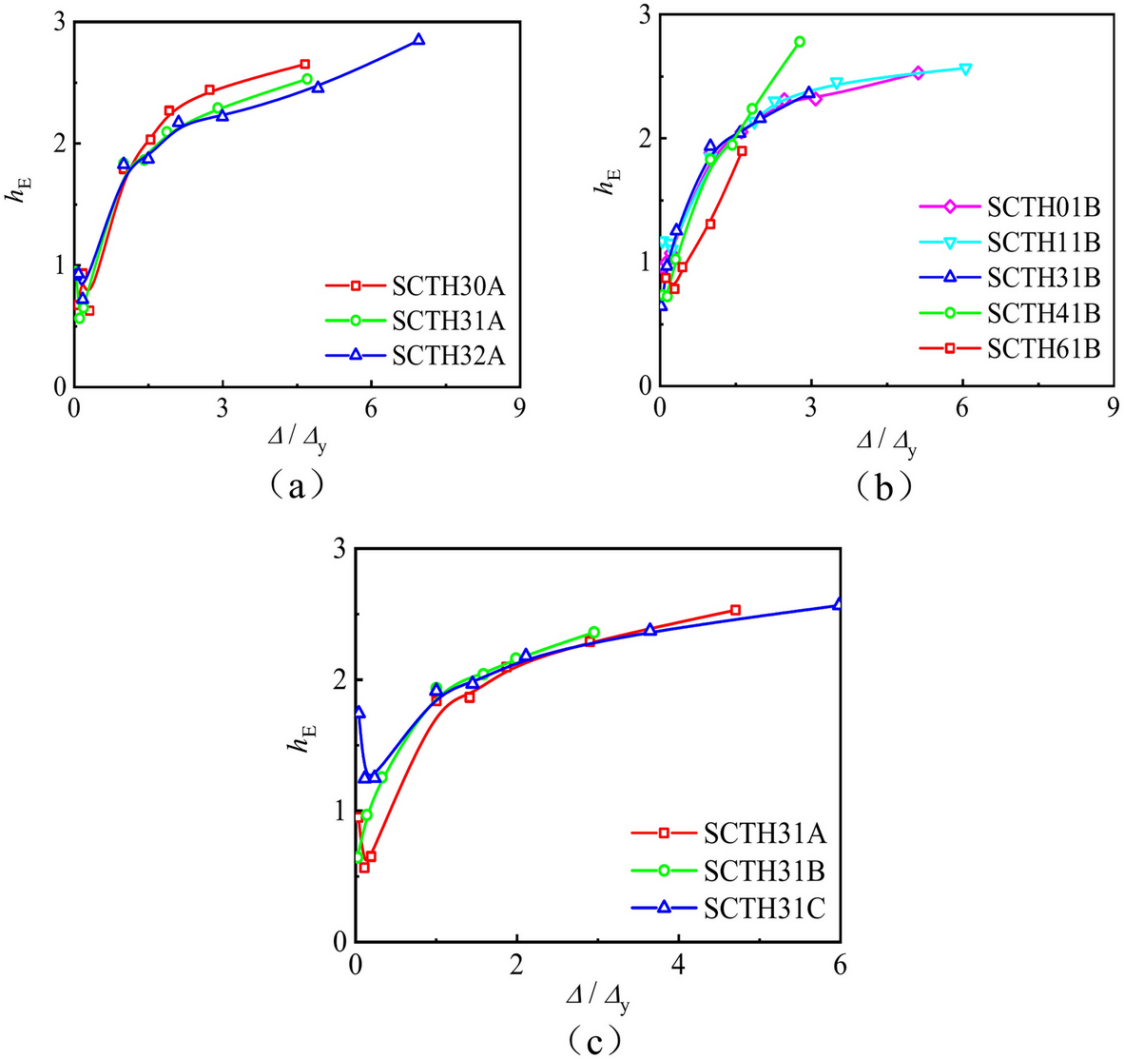
The energy dissipation curves of all specimens. (**a**) Specimens with different *m*_t_, (**b**) Specimens with different *n*. **c** Specimens with different *α*.

Energy dissipation(*h*_E_) refers to the ratio of energy dissipation within a vibration cycle to the elastic potential energy at the point of maximum amplitude. Figure 10 shows the energy dissipation curves of all specimens. It can be seen that the energy dissipation coefficient first decreases and then increases, and the specimen with a larger axial compression ratio has a faster increase in its energy dissipation coefficient. This indicates that the *n* is the main factor causing energy loss for concrete-filled square CFRP steel tube under compressive-torsional hysteresis loading.

## FE Model of circular section compressive-shear specimens

### Finite element modeling and parameter settings

ABAQUS 6.14 is used to obtain the performance of concrete-filled square steel tube with stub columns when subjected to compressive-torsional hysteresis loading. In current modeling, the nonlinearity of materials and geometry has been considered. Table 5 shows a typical finite element model. For each component material, steel and concrete, due to their material properties, elastic–plastic models for finite element analysis are used.

**Table 5:** Geometry of the specimens and mesh type.

Geometry	Steel	Concrete	CFRP	End plate
Section (mm)	120 × 120	114.8 × 114.8	Different dimensions	200 × 200
Thickness(mm)	2.6	–	Different dimensions	20
Length (mm)	Different	Different	Different	–
Type of geometry	Solid	Solid	Shell	Solid
Mesh type	C3D8R	C3D8R	M3D4	C3D8R

For each component material, steel and concrete, due to their material properties, elastic–plastic models for finite element analysis is used. Furthermore, the Von Mises stress criterion is selected for identifying the yield stage of the material^24–27^. To accurately simulate the interaction between the steel tube and its concrete core, a surface-to-surface contact model is utilized. This approach involves the application of hard contact and Coulomb friction theories to simulate the normal and tangential interactions, respectively. It’s important to note that when the friction coefficient falls below 0.25, the predictive accuracy for the descending portion of the load-deformation response curve in steel–concrete columns may be compromised; therefore, a coefficient of 0.6 has been strategically chosen. In this model, the inner surface of the steel tube is designated as the primary contact interface, and the surface of the concrete core is treated as the secondary interface. To precisely capture the dynamics of their interaction, the “limited sliding” setting is employed to define the contact behavior between these two structural components. Concrete has plastic damage coefficient and stiffness recovery coefficient under hysteresis loading. After extensive calculation, its parameters are determined to be: tensile plastic damage coefficient *b*_t_ is 0.6–0.85, and compressive plastic damage coefficient *b*_c_ is 0.8–0.95. The recovery coefficient of tensile stiffness *ω*_t_ is 0, and the recovery coefficient of compressive stiffness *ω*_c_ is 0.8. Loading control and graded loading are adopted and kept consistent with test, with loading of 0.25 *T*_uc_ (Estimated bearing torque), 0.5 *T*_uc_, and 0.7*T*_uc_ respectively, and each level of loading is cycled twice; Afterwards, displacement control and graded loading are adopted, with loading of 1.0 Δ_θ_ (The torsional angle corresponding to the yield of steel), 1.5 Δ_θ_, 2.0 Δ_θ_, 3.0 Δ_θ_, 5.0 Δ_θ_, and 7.0 Δ_θ_.

**Fig. 11:**
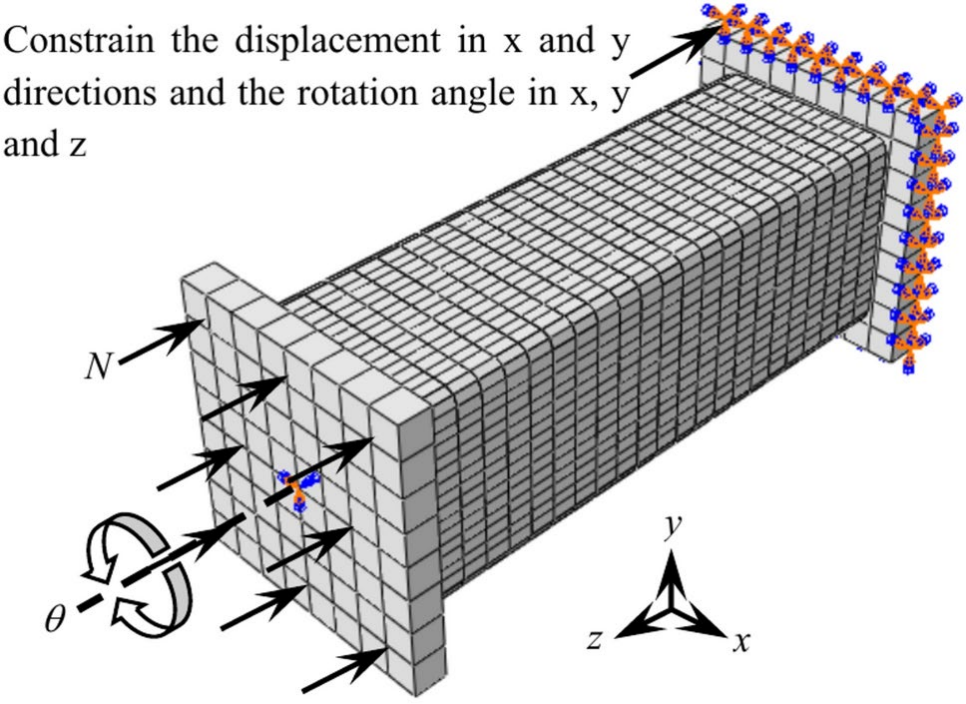
Boundary conditions of the specimens with stub columns when subjected to compressive- torsional hysteresis loading.

One side serves as a fixed end, constraining its displacement and rotation angle in the *x*, *y* and *z* directions ,and the other side set as the loading end. Apply the axial loading *N* is applied on the the loading end, and *N* remains constant throughout the entire loading process. Then, a reference point is set at the center of the end plate and coupled with the loading end plate, and turning angle *θ* is applied to the reference point to simulate the effect of torque. Boundary conditions of the specimens is shown in Fig. 11

### Verification of finite element models

1. *T*-*θ* hysteretic curve curves

**Fig. 12:**
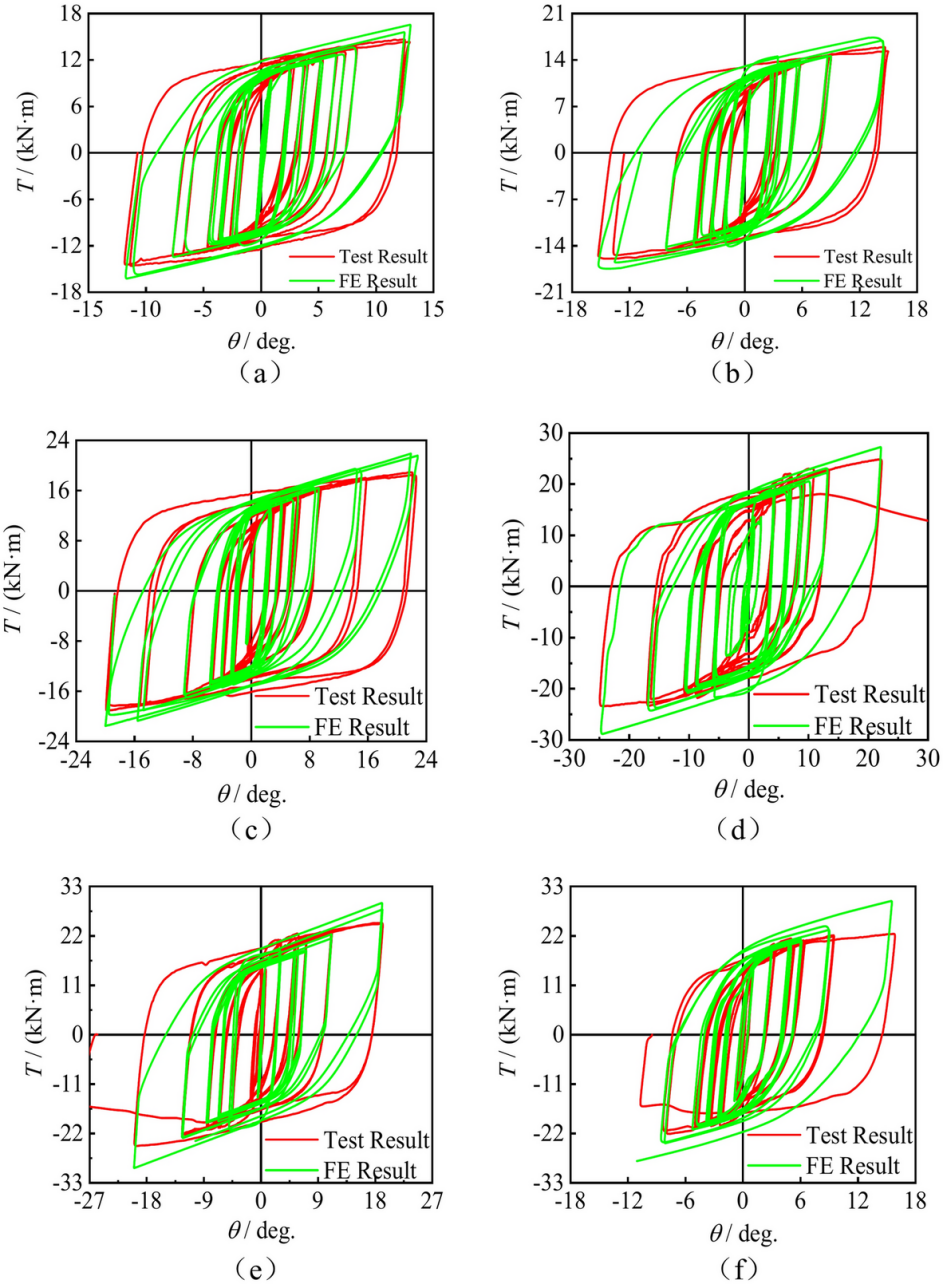

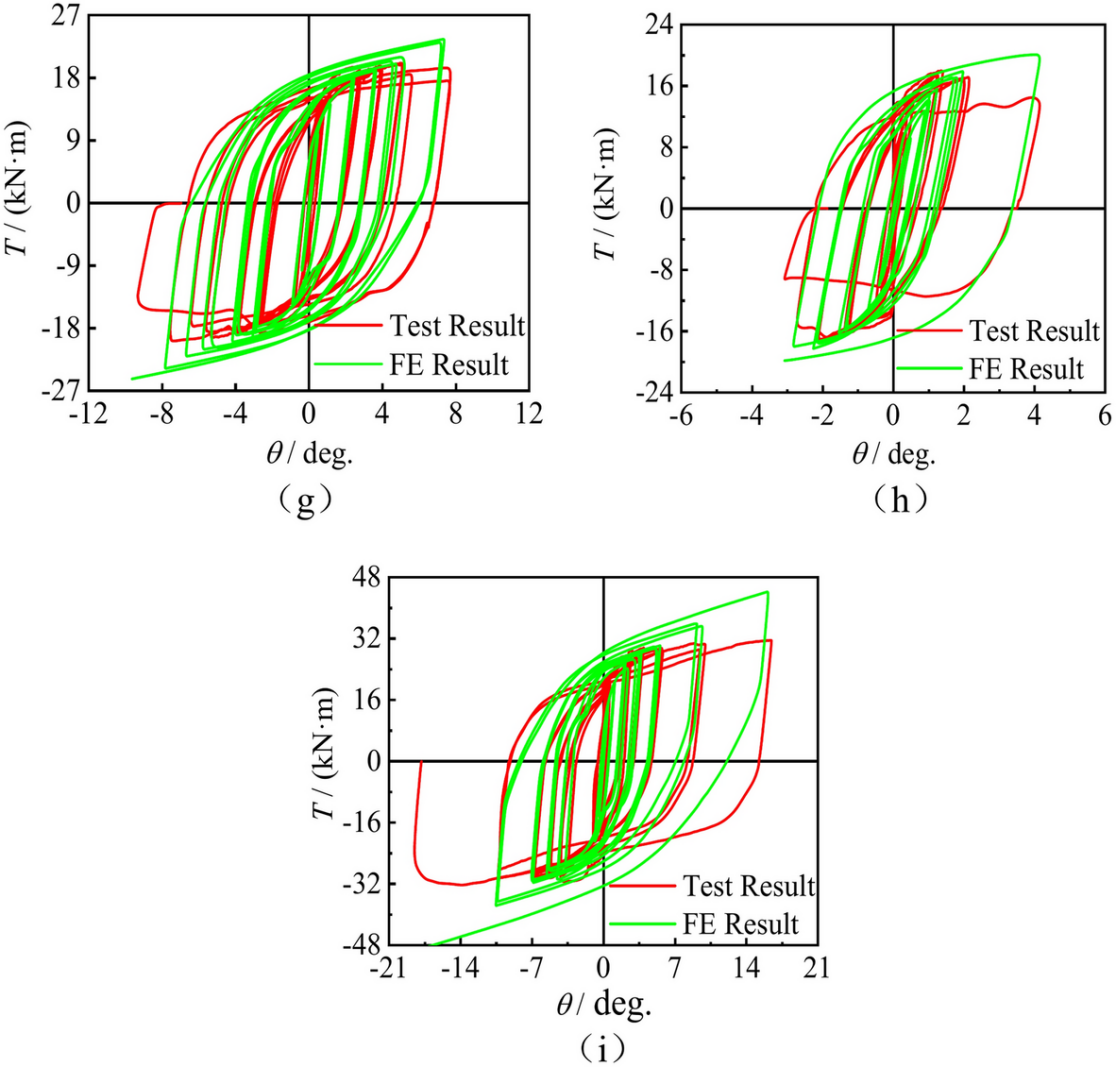
*T*-*θ* curves comparation of concrete-filled square steel tube with stub columns when subjected to compressive- torsional hysteresis loading. (**a**) SCTH30A, (**b**) SCTH31A, (**c**) SCTH32A, (**d**) SCTH01B, (**e**) SCTH11B, (**f**) SCTH31B, (**g**) SCTH41B, (**h**) SCTH61B, (**i**) SCTH31C.

Figure 12 presents a detailed comparison between the simulated and experimental *T*-*θ* response curves for short columns made of concrete-filled square CFRP steel tube compressive-torsional specimens under hysteresis loading, demonstrating the correlation between theoretical models and actual physical testing outcomes. The hysteresis curves calculated by the finite element models of each specimens can basically match the experimental curve and reflect the damage situation of the specimens under cyclic loading well during the loading and unloading stages, and all simulated hysteresis curves are spindle shaped without obvious pinching effect.

**Fig. 13:**
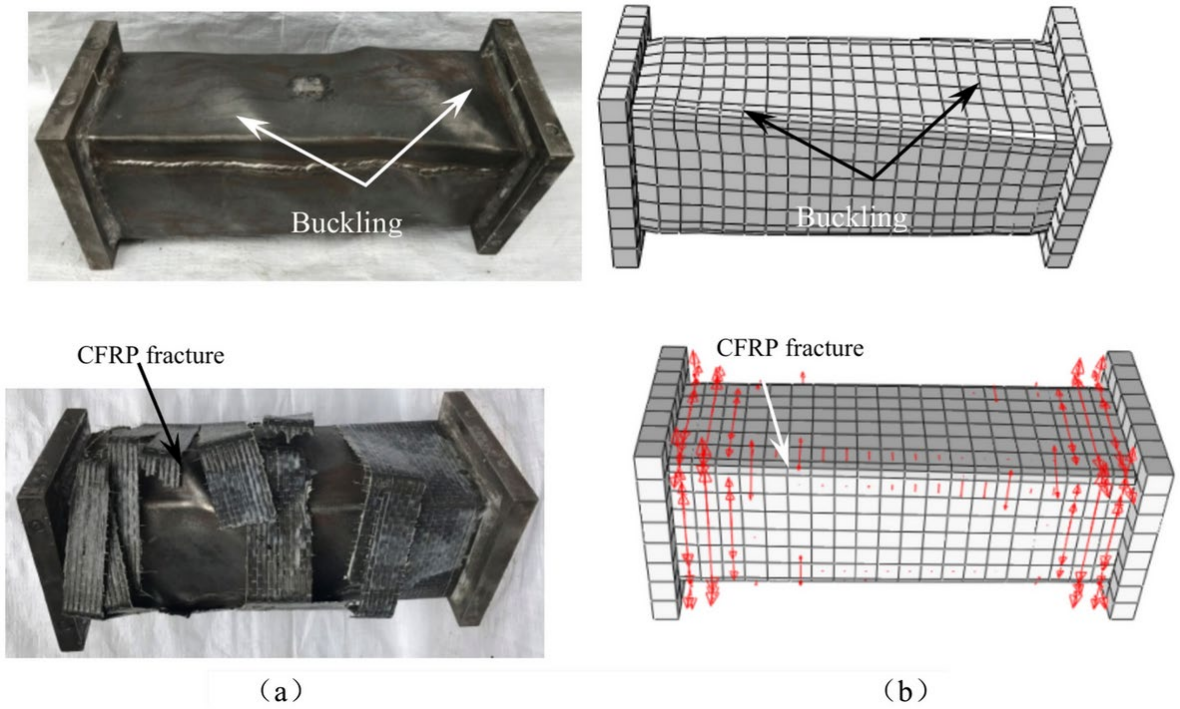
Failure mode comparation of concrete-filled square steel tube with stub columns when subjected to compressive- torsional hysteresis loading. (**a**) Test result, (**b**) FE result.

Figure 13 presents failure mode comparation of concrete-filled square steel tube with stub columns when subjected to compressive- torsional hysteresis loading. Through comparison, it was found that the local buckling of steel pipes in specimens without CFRP was more severe than in specimens with CFRP. In addition, the failure mode of the model established through finite element analysis is basically consistent with the experimental results, indicating that the simulation results are in good agreement with the experimental results.

## Main parameter analysis

### The impact of material strength

The parameter analysis section selected the most important parameters of steel reinforced concrete in practical engineering as the research object, including the number of CFRP layers (mt), yield strength of steel (fy), compressive strength of concrete (fcu), and the ratio of steel to concrete cross-sectional area (α = As/Ac).

**Fig. 14:**
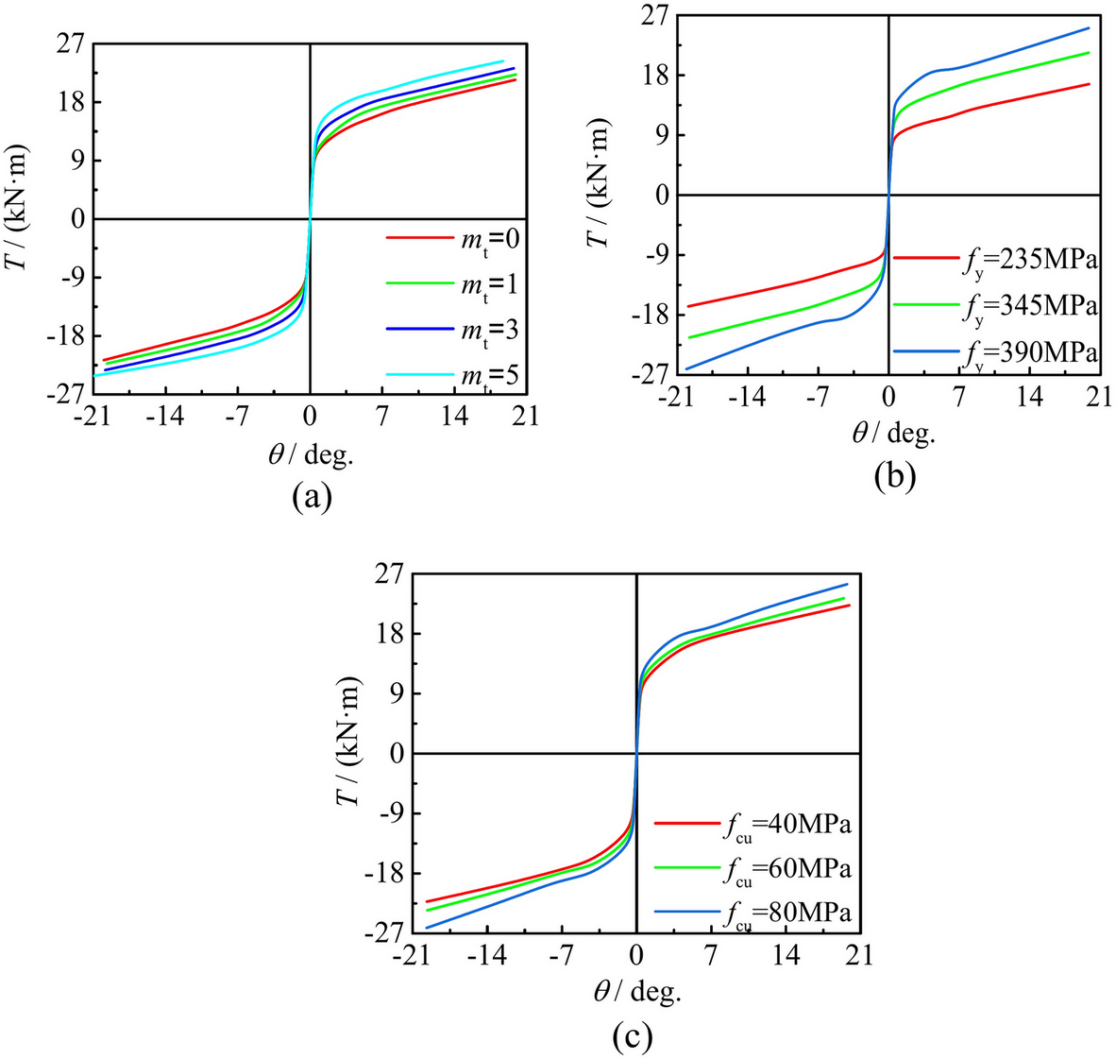
Effect of material parameters to square section specimens under compressive-torsional hysteresis loading. (**a**) Effect of *m*_t_ to square section specimens under compressive-torsional hysteresis loading, (**b**) Effect of *f*_y_ to square section specimens under compressive-torsional hysteresis loading, (**c**) Effect of *f*_cu_ to square section specimens under compressive-torsional hysteresis loading.

In Fig. 14a, the lifting of the CFRP layer has a significant effect on the *T*-*θ* skeleton curve response of the specimen subjected to compression torsion lag load. The results show that the bearing capacity of the curve is improved with the increase of *m*_t_. This result also fully reflects the proportional relationship between the number of carbon fiber cloth layers and the constraint effect enhancement, and verifies the fidelity of finite element analysis from another aspect.

Figure 14b and c illustrate the effect of changes in steel yield strength and concrete compressive strength on the* T*-*θ* skeleton curve profile of the specimen. As concrete and steel are the main constituent materials, their strength changes have a great impact on the overall mechanical properties of the specimen, so the strength improvement is positively correlated with the bearing capacity of the specimen.

### The impact of steel ratio and axial compression ratio

**Fig. 15:**
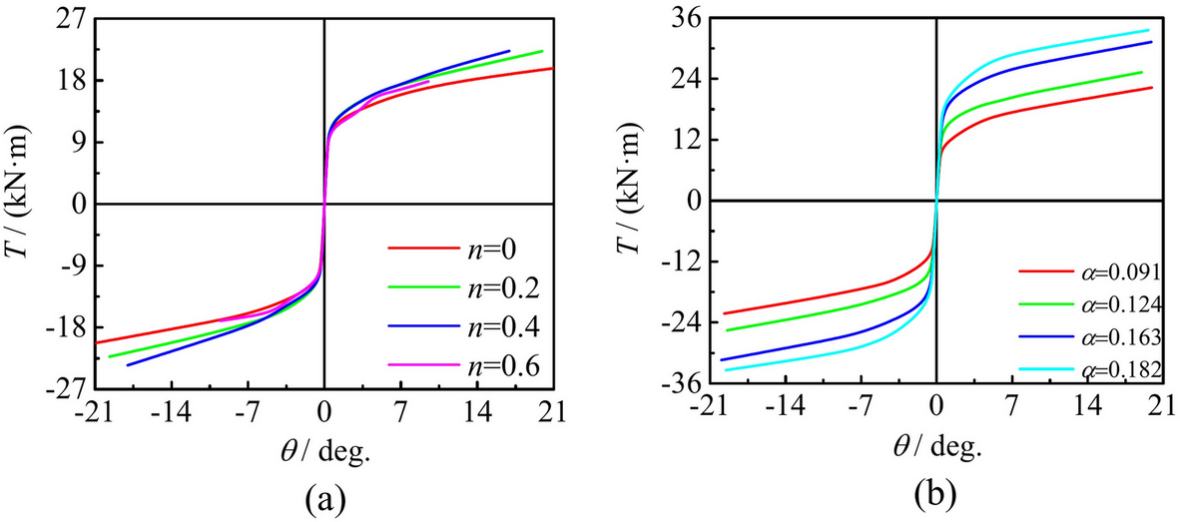
Effect of steel ratio and shear span ratio to square section specimens under compressive-torsional hysteresis loading. (**a**) Effect of *n* to square section specimens under compressive-torsional hysteresis loading, (**b**) Effect of *α* to square section specimens under compressive-torsional hysteresis loading.

Figure 15a illustrates the effect of changing α on the behavior of the *T*-*θ* skeleton curve of the sample. α not only improves the bearing capacity of the specimen, but also increases the stiffness of the elastic stage. Figure 15b describes the effect of axial compression ratio on *T*-*θ* skeleton curve characteristics. When n increases (0 < *n* ≤ 0.45), the bearing capacity and stiffness are gradually enhanced, but the change of parameters has no effect on the shape of skeleton curve. By contrast, as *n* continuous increases, the initial stiffness of the component begins to decrease, and the bearing capacity also decreases when* n* > 0.45. The shape of the skeleton curve begins to change, and a descending segment appears.

## Conclusion

1. The hysteresis curves are spindle shaped, relatively full, without any pinching phenomenon. Hysteresis curve approximately shows a linear variation in the initial stage of loading. The stiffness of the specimens gradually decreases after yielding. During the process from unloading to reverse loading, the stiffness of the specimens remain basically unchanged, and the loading of the specimen decreases in the later stage of loading. In addition, the strain curve in the 45° direction is always a hysteresis curve with alternating tensile and compressive states. The steel tube and CFRP can work well together.
2. The application of FE modeling techniques to analyze specimens under compressive-torsional hysteresis loading has demonstrated reliability, aligning closely with empirical data and the modes of failure observed during experimentation.
3. The results of parameter analysis indicate that concrete strength, steel strength, number of transverse CFRP layers, and steel ratio have no significant impact on the shape and stiffness of the hysteresis skeleton curve. With the increase of concrete strength, steel strength, and steel ratio, the bearing capacity of the component increases. The increase in the number of transverse CFRP layers results in a slight increase in the bearing capacity of the components. The steel ratio has no significant effect on the shape and initial stiffness of the hysteresis skeleton curve, but has a significant impact on the bearing capacity, which increases with the increase of steel ratio.

## Data Availability

The datasets used and/or analysed during the current study available from the corresponding author on reasonable request.

## List of symbols

*A*_c_: Cross-sectional area of concrete
*A*_cfl_: Cross-sectional area of longitudinal CFRP
*A*_cft_: Cross-sectional area of transverse CFRP
*A*_s_: Cross-sectional area of steel tube
*B*: Side-width of steel tube
CF-CFRP-ST: Concrete filled CFRP-steel tubes
CF-CFRP-T: Concrete filled CFRP tube
CFRP: Carbon fiber reinforced plastic
CFST: Concrete filled steel tube
CFT: Concrete filled tube
*E*_c_: Elastic modulus of concrete
*E*_cf_: Elastic modulus of carbon fiber sheet
*E*_s_: Elastic modulus of steel tube
*f*_cfl_: Ultimate tensile strength of longitudinal CFRP
*f*_cft_: Ultimate tensile strength of transverse CFRP
*f*^′^_cf_: Tensile strength of carbon fiber sheet
*f*_cu_: Compressive strength of cubic concrete
*f*_u_: Ultimate tensile strength of steel tube
*f*_y_: Yield strength of steel tube
*L*: Length of specimen
*m*_l_: Number of layer(s) of longitudinal CFRP
*m*_t_: Number of layer(s) of transverse CFRP
S-CF-CFRP-ST: Square concrete filled CFRP-steel tube
S-CFST: Square concrete filled steel tube
*T*: Torque
*W*_cfscm_: Flexural modulus of the member
*t*_cf_: Thickness of one-layer carbon fiber sheet
*t*_s_: Wall-thickness of steel tube
*v*_s_: Poisson’s ratio of steel tube
*ε*: Strain
*ε*_45_: Strain in 45° direction
*ε*_cf_: Strain of CFRP
*ε*^′^: Elongation percentage of steel tube
*ε*_cf45_: Strain of CFRP in 45° direction
*ε*_cfl_: Strain of Longitudinal CFRP
*ε*_cflr_: Rupture strain of longitudinal CFRP
*ε*_cft_: Strain of transverse CFRP
*ε*_cftr_: Rupture strain of transverse CFRP
*ε*_l_: Longitudinal strain
*ε*_s_: Strain of steel tube
*ε*_s45_: Strain in 45° direction of steel tube
*ε*_sl_: Longitudinal strain of steel tube
*ε*_st_: Transverse strain of steel tube
*ε*_t_: Transverse strain
*h*_E_: Energy dissipation
*θ*: Rotation angle
*ξ*_cf_: Confinement factor of transverse CFRP
*ξ*_s_: Confinement factor of steel tube
